# MpbPPI: a multi-task pre-training-based equivariant approach for the prediction of the effect of amino acid mutations on protein–protein interactions

**DOI:** 10.1093/bib/bbad310

**Published:** 2023-08-31

**Authors:** Yang Yue, Shu Li, Lingling Wang, Huanxiang Liu, Henry H Y Tong, Shan He

**Affiliations:** School of Computer Science from the University of Birmingham, UK; Centre for Artificial Intelligence Driven Drug Discovery at Macao Polytechnic University; Centre for Artificial Intelligence Driven Drug Discovery at Macao Polytechnic University; Centre for Artificial Intelligence Driven Drug Discovery at Macao Polytechnic University; Centre for Artificial Intelligence Driven Drug Discovery at Macao Polytechnic University; School of Computer Science, the University of Birmingham, Edgbaston, Birmingham, B15 2TT, UK

**Keywords:** protein binding affinity change prediction, equivariant neural network, multi-task pre-training, protein engineering

## Abstract

The accurate prediction of the effect of amino acid mutations for protein–protein interactions (PPI $\Delta \Delta G$) is a crucial task in protein engineering, as it provides insight into the relevant biological processes underpinning protein binding and provides a basis for further drug discovery. In this study, we propose MpbPPI, a novel multi-task pre-training-based geometric equivariance-preserving framework to predict PPI  $\Delta \Delta G$. Pre-training on a strictly screened pre-training dataset is employed to address the scarcity of protein–protein complex structures annotated with PPI $\Delta \Delta G$ values. MpbPPI employs a multi-task pre-training technique, forcing the framework to learn comprehensive backbone and side chain geometric regulations of protein–protein complexes at different scales. After pre-training, MpbPPI can generate high-quality representations capturing the effective geometric characteristics of labeled protein–protein complexes for downstream $\Delta \Delta G$ predictions. MpbPPI serves as a scalable framework supporting different sources of mutant-type (MT) protein–protein complexes for flexible application. Experimental results on four benchmark datasets demonstrate that MpbPPI is a state-of-the-art framework for PPI $\Delta \Delta G$ predictions. The data and source code are available at https://github.com/arantir123/MpbPPI.

## INTRODUCTION

Protein–protein interactions (PPIs) play a crucial role in many biological processes, including antibody–antigen binding, cell apoptosis and signal transduction [1–3]. Alterations in PPIs can affect the formation of multi-protein complexes, causing disruption to both intercellular communication networks and intracellular signaling pathways, ultimately contributing to the development of diseases such as cancer and drug resistance [4]. Amino acid (AA) mutations are a primary cause of PPI alterations, with different mutations leading to different disturbances in PPIs, and resulting in different phenotypic outcomes. Understanding the potential mechanisms by which AA mutations impact PPIs is vital for developing therapies that target these interactions [5–8]. Although various experimental methods are available to identify the effects of mutations, they are often costly and time consuming. To overcome this challenge, fast and reliable computational alternatives are necessary [9].

The change in binding affinity caused by AA mutations, or $\Delta \Delta G$, represents the difference in binding affinity between mutant-type (MT) and wild-type (WT) protein complexes [10]. Over the years, numerous computational approaches have been developed to predict $\Delta \Delta G$ [11]. Early $\Delta \Delta G$ tools mainly employed physical energies as features to describe PPIs, such as FoldX [12], Rosetta [13] and BeAtMuSiC [14]. These methods rely on a (linear) combination of physical energies dominated by van der Waals interaction, hydrogen bond, electrostatic interaction, solvation, etc. terms. However, many of these methods faced several limitations, including insufficient conformation sampling in the mutation region and conformational efficiency [15].

In recent years, the rapid expansion of experimental data and computational resources led to the emergence of machine learning methods, especially those mainly focusing on characterizing geometric properties of protein structures [16]. The structure of protein dictates its biochemical function [17], thus the combination of fully captured protein structural characteristics and the powerful non-linear fitting capability of machine learning methods could provide a more accurate approximation of PPI $\Delta \Delta G$ [18]. For instance, aiming to tackle $\Delta \Delta G$ predictions caused by AA single-point mutations, Geng et al. [19] developed a random forest-based algorithm iSEE to process PPI interface structure features enhanced by evolutionary conservation and energy-based information. Based on the specially designed graph-based structural signatures representing the residue environment, an online $\Delta \Delta G$ analysis webserver named mCSM-PPI2 was built by Rodrigues et al. [18]. Furthermore, Wang et al., Liu et al. and Wee et al. proposed their algebraic topology-based methods, respectively, to produce the simplified physical structural representations of protein–protein complexes with the consideration of AA site mutation information. The generated representations were fed into different ensemble decoders to calculate the final $\Delta \Delta G$ [1, 20, 21].

In addition to traditional machine learning methods, the deep learning-based geometric method graph neural network (GNN) could be a more efficient way for extracting structural representations of protein–protein complexes. This is because it can directly take the geometric graph which can be easily constructed from raw protein coordinates as model inputs [22]. A typical processing pipeline for such methods involves acquiring a protein–protein complex graph after AA site mutations, then inputting the WT and mutant protein–protein complex graphs into a GNN to obtain the respective graph structure representations. The $ \Delta \Delta G $ is then predicted by comparing these two representations using an independent decoder. However, due to the difficulty in acquiring mutant complex structure ground truth, corresponding simulated structures or assumed proxies are usually needed. For example, Liu et al. [15] cropped the original atom-level WT and computationally simulated mutant complex graphs based on a pre-defined distance threshold. They then input these cropped graphs into a pre-trained GNN encoder, to produce the final low-dimensional representation that captures the structural difference between WT and mutant complexes. Jiang et al. [22] designed an end-to-end GNN framework named DGCddG which takes assumed WT and mutant graphs equipped with multiple biochemical features for $ \Delta \Delta G $ predictions.

Most machine learning and deep learning methods currently rely on a combination of energy-based and structural-based features to predict the $\Delta \Delta G$ value associated with a given PPI mutation [18]. However, energy-based features can be computationally expensive, while many existing structural-based features focus on modeling single residue-wise contact relationships or inter-atom interactive relationships, which may not capture more complete geometric relationships between the backbone, side chains and their interactions within AA residues. The relative positions and orientations of residues, as well as the interactions between their side chain and backbone atoms, can significantly impact the alignment of amino acids within a protein structure and influence protein–protein interactions.

Moreover, transfer learning, which aims to capture general regulations of downstream samples through pre-training the model on more accessible other data (not involving the type of labels in downstream tasks) [23–25], can help overcome the challenge of limited experimental mutation datasets for $\Delta \Delta G$ prediction. It is crucial to ensure that the pre-training dataset is carefully selected and prevent unintended information leakage to downstream tasks, which could result in overoptimistic model evaluation results. Meanwhile, when predicting PPI $\Delta \Delta G$ using machine learning or deep learning algorithms, it is also important to consider the impact of amino acid mutations on protein structure [15]. Mutant structures are able to provide valuable insights into how the amino acid substitution affects the protein structure, which can serve as input to these algorithms to predict the $\Delta \Delta G$. Many methods are available for generating the mutant structures, including empirical models (FoldX [12]), homology modeling (MODELLER [26], SwissModel [27]) and deep learning methods (AlphaFold2 [28]), among others. Each method has its strengths and limitations, and the choice of method can affect the accuracy of the mutant structure and, subsequently, the $\Delta \Delta G$ prediction. Therefore, it is crucial to carefully evaluate and compare different methods and choose the most appropriate method for a given PPI system.

To summarize, a more efficient and sufficient characterization and learning of comprehensive geometric relationships within PPI systems (in which the mutant structures are unknown), under insufficient labeled PPI $\Delta \Delta G$ data, is needed. We solved these issues by introducing a flexible geometric equivariant graph neural network framework MpbPPI, for the precise prediction of PPI $\Delta \Delta G$ resulting from single and multiple missense mutations. Specifically, to capture more comprehensive PPI geometric information, we designed two types of residue-level contact graphs that include the different-scale geometric interactive relationships between residues, between side chain atoms and between side chain atoms and residue backbone atoms. To better utilize unlabeled PPI structures to produce low-dimensional protein–protein complex representations with good generalization for limited number of downstream samples, we first proposed a geometric equivariant encoder (GEE) contained in MpbPPI to encode the above contact graphs, and it will learn effective PPI geometric regulations through our specially designed multitask pre-training technique performed on a strictly screened pre-training dataset. Furthermore, MpbPPI serves as a framework compatible with multiple mutant generation tools, enabling flexible handling of mutant structures including ones led by multiple missense mutations.

We conducted extensive experiments on MpbPPI to evaluate its performance in conjunction with three distinct mutation structure generation tools: FoldX, MODELLER and AlphaFold2. Our approach achieved state-of-the-art performance on three single mutation datasets and one more challenging multiple mutations dataset under two realistic evaluation settings. The results demonstrate that MpbPPI is a promising approach for predicting protein–protein binding affinity alterations caused by AA mutations.

The rest of the paper is organized as follows. We first provide the overall description of our proposed framework in Overview of MpbPPI section. The details of our experiments are given in the Results section. The analysis about the extension and flexibility of MpbPPI is elucidated in the Discussion section, and the detailed model implementation information is laid out in the Methods section.

## RESULTS

### Overview of MpbPPI

**Figure 1:**
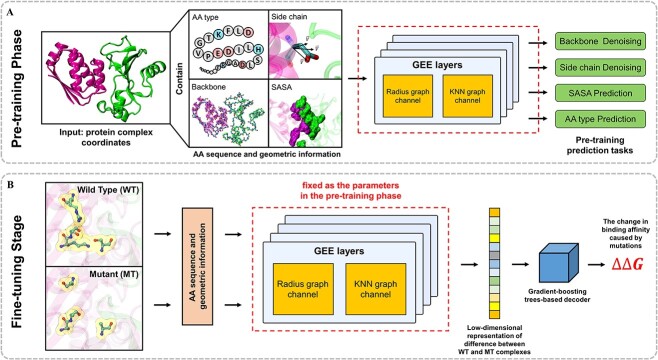
The flowchart of the MpbPPI framework. For each pre-training and downstream sample point, MpbPPI generates the residue-level KNN and radius contact graphs, which contain different-scale residue backbone and side chain geometric information of the corresponding protein–protein complex structure (see Methods section for details). In the pre-training phase (**A**), the proposed GEE encoder learns the geometric regulations of protein–protein complexes through our defined four geometric property-related denoising/recovery tasks. After that, MpbPPI uses a GBT-based decoder to predict PPI $\Delta \Delta G$ for a WT–MT complex pair based on their encoded representations (**B**).

As an overview (Figure 1), MpbPPI fully learns the geometric regulations of protein–protein complexes based on the pre-training dataset (composed of protein complexes sharing both a low topological similarity and sequence identity to the complexes in the downstream datasets, details on the data collection can be found in Supplementary Material), by first generating two types of residue-level contact graphs that characterize different inter-residue geometric interactive relationships. Specifically, for each protein–protein complex in our strictly screened pre-training dataset, MpbPPI creates both a residue-level $\mathrm{K}$-nearest neighbor (KNN) and radius contact graphs that contain both residue backbone and side chain atom-related geometric information (see Methods section for details). The graph structure of two contact graphs is used to model the relatively large-scale residue-level geometric relationships, while the residue backbone and side chain atom geometric information within it (as residue graph node features) are used to sufficiently capture the relatively small-scale complex interactive relationships between various atoms in a single residue.

Next, to better facilitate the learning of geometric regulations of the PPI structures, we devised four pre-training tasks (to be performed simultaneously) for the protein geometric equivariant encoder (GEE) of MpbPPI, including (residue) backbone denoising, side chain denoising, solvent-accessible surface area (SASA) prediction and AA type prediction. Intuitively, multiple pre-training objectives aiming at learning geometric properties of individual residues themselves from different perspectives will encourage the GEE to capture the comprehensive interactive relationships within a complex, which is ultimately beneficial to generate high-quality PPI structural representations for downstream analyses. Based on this intuition, we corrupted the contact graphs of each pre-training protein–protein complex by adding noise to the 3D coordinates of the randomly chosen residue backbone and side chain atoms, and the corrupted graphs were sent to the GEE to perform the multi-task pre-training.

**Figure 2:**
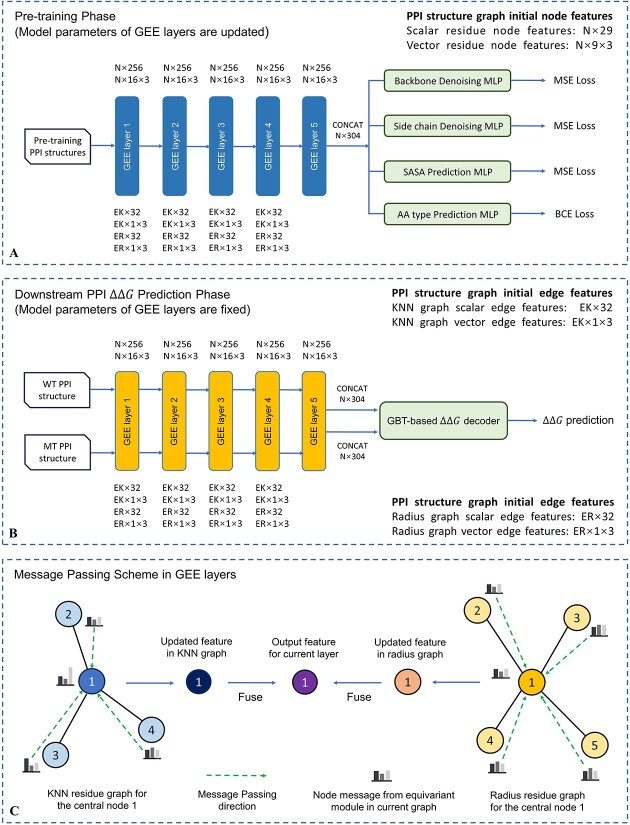
Panel (**A**) illustrates the MpbPPI data flow in the pre-training phase. In this phase, the pre-training protein–protein complex (residue number: N) represented by the KNN (edge number: EK) and radius contact graphs (edge number: ER) is sent to a five-layer GEE encoder. Based on message propagation, the encoder outputs updated embeddings of every residue node in current complex, which will be sent to four multi-layer perceptions (MLPs) specific to different pre-training tasks simultaneously, for guiding the model optimization. The input/output dimensions of each intermediate layer are shown around this layer. For downstream $\Delta \Delta G$ prediction phase (**B**), WT and mutant PPI structures represented by the same type of contact graphs as above are sent to the trained GEE to produce separate residue node embedding sets, which are then sent to the GBT-based decoder to predict the final $\Delta \Delta G$ for current sample point (see Methods section). Panel (**C**) illustrates the basic message propagation scheme in each GEE layer, in which the similar operations will be performed to each (central) residue node in the protein–protein complex.

After the pre-training, MpbPPI first generates the aforementioned contact graphs for the WT and corresponding mutant complexes (which are from various mutant generation tools) of each sample point in the downstream $\Delta \Delta G$ dataset. Based on employing message passing to propagate different-scale geometric information within the contact graphs (Figure 2C), GEE encoder produces the low-dimensional structural representation for comparing the geometric difference between WT and mutant complexes. Finally, MpbPPI employs the gradient-boosting trees (GBT), which is an ensemble predictor specialized at handling overfitting, to predict the PPI $\Delta \Delta G$ for a WT–MT complex pair from the generated representation.

The overall diagram further illustrating the data flow of MpbPPI and how its components are linked is shown in Figure 2, and more details and explanations of the whole MpbPPI framework can be found in the Methods section.

### MpbPPI yields accurate PPI $\Delta \Delta \boldsymbol{G}$ prediction

To comprehensively compare MpbPPI with other existing advanced approaches, we considered three single AA site mutation benchmark datasets, including single-point mutations in the AB-Bind dataset [29] (S645), non-redundant interface single-point mutations in the SKEMPI dataset [11] (S1131) and single-point mutations in the SKEMPI2 dataset [18] (S4169). We also incorporated one more challenging benchmark dataset that contains multiple-point AA mutations (as multiple-point mutations could bring more complex PPI system conformation changes compared with single-point mutations): complete sample points of the AB-Bind dataset [30], denoted as M1101.

#### Mutation-level cross-validation results

Based on the above four datasets, following the convention, we first performed the 10-fold cross-validation splitting data based on mutation sample points and used mean Pearson’s correlation coefficient (${R}_{\mathrm{P}}$) as the main evaluation metric [1]. In other words, all mutation sample points in each dataset will be uniformly split into 10 folds for the 10-fold cross-validation. In addition, for each dataset, we ran such 10-fold cross-validation five times independently. For each repeat, the whole dataset was randomly shuffled to make different mutation sample points enter each fold, and we reported the average results of these independent runs. Under this evaluation setting, in order to examine the performance of MpbPPI based on different mutant structure generation tools, we created three variants of MpbPPI with different suffixes indicating the mutant complex source (i.e. MpbPPI-FoldX, MpbPPI-MODELLER, MpbPPI-AlphaFold2, the detailed description of mutant complex generation is in Supplementary Material), and compared them with seven baseline methods for PPI $\Delta \Delta G$ predictions, including GeoPPI [15], TopNetTree [1], DGCddG [22], PerSpect-EL [21], FoldX [12], Rosetta macromolecular modeling suite (Flex ddG) [31] and MM/GBSA [32–34]. The former four methods are representative advanced geometric-based machine learning methods, while the latter three are mainstream energy-based methods. The detailed description of these methods can be found in Supplementary Material.

Our experimental results are shown in Table 1, in which the results of GeoPPI [15], TopNetTree [1], DGCddG [22] and PerSpect-EL [21] were obtained from the original papers. We also reported more experimental results, including the root-mean-square error (RMSE) and mean absolute error (MAE) of our method and additional analysis, in Supplementary Table 2 and Supplementary Figures 1 and 2. In summary, based on the five times of independent runs of the 10-fold cross-validation, MpbPPI (MpbPPI-FoldX) achieved overall better performance compared with other competitive methods, while only on the S645 dataset, the second-best result was achieved. Another important advantage of MpbPPI is that it can tackle multiple-point mutation cases more easily because of its flexibly direct input of the mutant structure (graph) from various sources.

**Table 1:** Comparison of the ${R}_{\mathrm{P}}$ of the baseline methods on downstream PPI $\Delta \Delta G$ datasets (the results of GeoPPI [15], TopNetTree [1], DGCddG [22], PerSpect-EL [21] are the 10-fold cross-validation results reported in their original papers)

S645	${\boldsymbol{R}}_{\mathbf{P}}$	M1101	${\boldsymbol{R}}_{\mathbf{P}}$
MpbPPI-FoldX	0.615 ± 0.013	MpbPPI-FoldX	**0.787 ± 0.002**
MpbPPI-MODELLER	0.598 ± 0.006	MpbPPI-MODELLER	0.780 ± 0.001
MpbPPI-AlphaFold2	0.577 ± 0.015	MpbPPI-AlphaFold2	0.765 ± 0.005
DGCddG	$\hbox{--}$	DGCddG	$\hbox{--}$
PerSpect-EL	0.590	PerSpect-EL	$\hbox{--}$
GeoPPI	**0.650**	GeoPPI	0.780
TopNetTree	**0.650**	TopNetTree	$\hbox{--}$
FoldX	0.271	FoldX	0.273
Flex ddG	0.067	Flex ddG	0.059
MM/GBSA	0.039	MM/GBSA	0.018
**S1131**	${\boldsymbol{R}}_{\mathbf{P}}$	**S4169**	${\boldsymbol{R}}_{\mathbf{P}}$
MpbPPI-FoldX	**0.865 ± 0.003**	MpbPPI-FoldX	**0.795 ± 0.004**
MpbPPI-MODELLER	0.847 ± 0.002	MpbPPI-MODELLER	0.782 ± 0.005
DGCddG	0.848	DGCddG	$\hbox{--}$
PerSpect-EL	0.853	PerSpect-EL	$\hbox{--}$
GeoPPI	0.850	GeoPPI	0.780
TopNetTree	0.850	TopNetTree	0.790
FoldX	0.423	FoldX	0.326
Flex ddG	0.217	Flex ddG	0.136
MM/GBSA	0.303	MM/GBSA	0.164

The bold data indicate the best result under current evaluation metric and dataset. Due to the high computational cost of AlphaFold2 (the computational cost analysis of the mutant generation tools is provided in Discussion), we tested performance of MpbPPI-AlphaFold2 based on the relatively smaller S645 and M1101 datasets.

In addition, for MpbPPI (MpbPPI-FoldX), when compared with GeoPPI, another geometric property pre-training-based method, under the strict pre-training sample screening and the same GBT predictor (see Methods), MpbPPI still overall outperformed it on the relatively larger single- and multiple-mutation datasets. This further demonstrated the effectiveness of the proposed different-scale geometric encoding framework and corresponding pre-training tasks. Interestingly, MpbPPI-FoldX consistently outperformed MpbPPI-MODELLER and MpbPPI-AlphaFold2, which indicated that under current PPI $\Delta \Delta G$ prediction benchmark datasets, FoldX could be a more appropriate tool as the source of mutant PPI protein–protein structures (the further analysis about these generation tools are provided in Discussion). Furthermore, in Supplementary Material, we provided extra experimental results about (i) directly correlating the difference between sequence representations of the mutant and wild-type complexes to the PPI $\Delta \Delta G$ value, and (ii) reproducing results of some representative baseline methods based on available information (Supplementary Table 1).

#### The effectiveness of devised geometric characteristics and their pre-training tasks

Based on the best-performing model MpbPPI-FoldX, under the same experimental and evaluation setting as the last section, we conducted the ablation study to discuss the effectiveness of our devised geometric characteristics and corresponding pre-training tasks. Specifically, we compared different variants of MpbPPI-FoldX, including MpbPPI (Backb+AA), MpbPPI (Backb+Sidec+AA), MpbPPI (Backb+SASA+AA) and MpbPPI (Backb+Sidec+SASA+AA), in which the suffixes represent different combinations of the used geometric characteristics plus their pre-training techniques (in other words, each suffix represents the presence of the corresponding residue node feature and pre-training task). Besides, to demonstrate the effectiveness of the auxiliary AA prediction task, we removed this prediction task from MpbPPI (Backb+Sidec+SASA+AA) and also put this variant into the comparison [denoted as MpbPPI (w/o AA)]. The evaluation results of these variants on each dataset are shown in Table 2 (we also reported the RMSE and MAE results in Supplementary Table 3).

**Table 2:** Comparison of the ${R}_{\mathrm{P}}$ of five MpbPPI-FoldX variants on each downstream dataset (the results of MpbPPI-FoldX variants were reported based on the average of five times of the independent 10-fold cross-validation executions)

S645	${\boldsymbol{R}}_{\mathbf{P}}$
MpbPPI (Backb+Sidec+SASA+AA)	**0.615 ± 0.013**
MpbPPI (Backb+SASA+AA)	0.575 ± 0.016
MpbPPI (Backb+Sidec+AA)	0.549 ± 0.008
MpbPPI (Backb+AA)	0.507 ± 0.016
MpbPPI (w/o AA)	0.613 ± 0.013
**M1101**	${\boldsymbol{R}}_{\mathbf{P}}$
MpbPPI (Backb+Sidec+SASA+AA)	**0.787 ± 0.002**
MpbPPI (Backb+SASA+AA)	0.761 ± 0.007
MpbPPI (Backb+Sidec+AA)	0.755 ± 0.005
MpbPPI (Backb+AA)	0.738 ± 0.003
MpbPPI (w/o AA)	0.785 ± 0.002
**S1131**	${\boldsymbol{R}}_{\mathbf{P}}$
MpbPPI (Backb+Sidec+SASA+AA)	0.854 ± 0.002
MpbPPI (Backb+SASA+AA)	**0.865 ± 0.003**
MpbPPI (Backb+Sidec+AA)	0.848 ± 0.003
MpbPPI (Backb+AA)	0.838 ± 0.003
MpbPPI (w/o AA)	0.831 ± 0.003
**S4169**	${\boldsymbol{R}}_{\mathbf{P}}$
MpbPPI (Backb+Sidec+SASA+AA)	0.793 ± 0.003
MpbPPI (Backb+SASA+AA)	**0.795 ± 0.004**
MpbPPI (Backb+Sidec+AA)	0.775 ± 0.003
MpbPPI (Backb+AA)	0.766 ± 0.004
MpbPPI (w/o AA)	0.766 ± 0.003

The bold data indicates the best result under current evaluation metric and dataset.

From the results we found that, compared with MpbPPI (Backb+AA) that did not use any explicit characteristics depicting geometric relationships between side chain atoms and between side chain and residue backbone atoms, the inclusion of side chain denoising and SASA prediction pre-training tasks (and corresponding features, see Methods) i.e. MpbPPI (Backb+Sidec+AA) and MpbPPI (Backb+SASA+AA) improved the performance. This clearly demonstrated the importance of our designed two types of side chain-related geometric learning pre-training tasks (and corresponding features). Besides, the performance margin between MpbPPI (w/o AA) and MpbPPI (Backb+Sidec+SASA+AA) illustrates the effectiveness of the auxiliary AA prediction task. Furthermore, as for the best-performing combination under the current experimental setting, it could vary for different datasets e.g. the combination of Backb+SASA+AA achieved the best result on S4169, but for S645, M1101 and S1131, the best combinations were Backb+Sidec+SASA+AA, Backb+Sidec+SASA+AA and Backb+Sidec+AA, respectively.

#### The prediction results in the more challenging test scenario

In this section, we further tested the generalization capability of MpbPPI through a WT protein–protein complex type-based data splitting setting. Specifically, to better examine the model performance on previously unseen WT protein–protein complexes (the training and test sample points in the above mutation-level cross-validation may share relatively more WT protein–protein complex types), we devised a data-splitting method in which original sample points were split into five folds and there was no intersection of WT protein–protein complex types between any of the two folds. We strived to make the both sample total number and WT protein–protein complex type number assigned to each fold to be as close as possible. Furthermore, we performed 5-fold cross-validation based on this data splitting scheme, in this case, the model cannot see any WT protein–protein complexes (types) in the test set during the training phase, which enables model performance examination in the more realistic application scenario. We performed such data splitting five times, for each repeat, making different WT protein–protein complex types enter each fold, and we reported the averaging results of these five times of independent (5-fold) cross-validation runs. A detailed description of the above data splitting can be found in Supplementary Material. For performance comparison, we considered available GeoPPI, FoldX, Flex ddG and MM/GBSA. For GeoPPI, for a fair comparison, we used its original released pre-trained encoder and GBT hyper-parameters on S4169 to reproduce the results on the S4169 dataset based on the same data splitting. In addition, to investigate the better MpbPPI setting in this challenging scenario, we included both MpbPPI (Backb+Sidec+SASA+AA) and MpbPPI (Backb+SASA+AA) since they achieved the best results on different downstream datasets in the last section.

**Figure 3:**
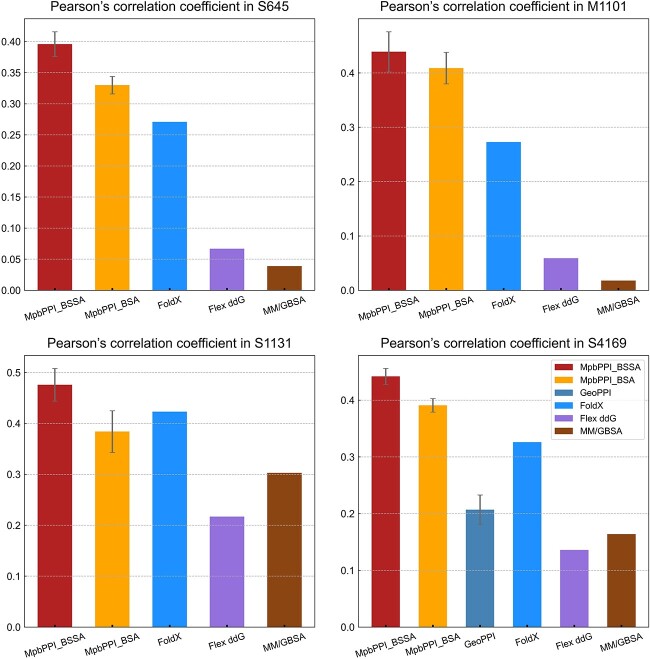
MpbPPI outperformed other involved methods for PPI $\Delta \Delta G$ prediction under the five-time WT protein–protein complex-based cross-validations. We reported the experimental results on each dataset based on the main evaluation metrics ${R}_{\mathrm{P}}$. For the machine learning-based methods, the results were expressed as mean ± SD, while for the empirical energy-based methods, the results were expressed as the mean value. MpbPPI (Backb+Sidec+SASA+AA) and MpbPPI (Backb+SASA+AA) were abbreviated as MpbPPI_BSSA and MpbPPI_BSA.

Our comparative study showed that [see Figure 3, in which MpbPPI (Backb+Sidec+SASA+AA) and MpbPPI (Backb+SASA+AA) were abbreviated as MpbPPI_BSSA and MpbPPI_BSA separately; the RMSE and MAE results are reported in Supplementary Table 4], MpbPPI exhibited more accurate predictive capability when facing previously unseen WT protein–protein complexes, which can further demonstrate the effectiveness of our method. Furthermore, we observed that MpbPPI_BSSA outperformed MpbPPI_BSA on all downstream datasets (the performance margins were 20, 7.3, 24 and 13% on S645, M1101, S1131 and S4169 for ${R}_{\mathrm{P}}$, respectively), indicating that the integration of a combination of the two devised side chain-related geometric characteristics can bring better model generalization in current task setting. Based on this, we recommend adopting MpbPPI (Backb+Sidec+SASA+AA) in an actual application setting.

## DISCUSSION

Mutations can alter the formation of protein complexes by enhancing or inhibiting interactions between proteins. This, in turn, affects various functions in the body, including catalyzing chemical reactions, transporting molecules and transmitting signals. Gaining insights into how mutations influence protein–protein interactions can help us understand the development and progression of diseases, ultimately leading to the creation of more effective treatments (the further example can be found in the illustrative example of the predicted outcomes of MpbPPI section of the Supplementary Material).

Computational modeling of changes in binding free energy ($\Delta \Delta G$) resulting from mutations allows for the prediction and manipulation of PPIs on a large scale. In this study, to better learn complete geometric regulations for limited numbers of downstream PPI $\Delta \Delta G$ samples from the proper pre-training dataset, we established a novel pre-training-based computational framework MpbPPI. MpbPPI can effectively evaluate the impact of single and multiple missense AA mutations on PPI binding affinity based on more comprehensive and computationally inexpensive geometric characteristics and flexible adapt to multiple mutant generation tools, which could provide valuable insights into the potential consequences of mutations on protein–protein interactions.

Recent advances in the PPI $\Delta \Delta G$ prediction methods, particularly the geometric GNN-based methods [15, 22], have significantly improved the predictive accuracy and stability. However, many methods focus on modeling single residue-wise contact relationships or inter-atom interactive relationships, omitting more comprehensive geometric relationships between different constituents in protein–protein complexes. Our MpbPPI addresses this issue by propagating specially designed different-scale geometric interactive information between residues, between side chain atoms, and between side chain and residue backbone atoms based on a new geometric equivariant message passing network. In addition, the scarcity of labeled PPI $\Delta \Delta G$ data presents a significant challenge. To overcome it, based on a strictly screened pre-training set for avoiding potential information leakage, we proposed four pre-training tasks to be performed simultaneously, which are compatible with the above different-scale geometric information, to improve MpbPPI’s generalization to unseen downstream samples. The robust predictive ability of the proposed geometric characteristics learning framework and corresponding pre-training tasks has been extensively validated on four benchmark datasets and compared with seven different methods under two realistic evaluation settings (the further discussion of some other optional features is provided in Supplementary Material).

Furthermore, accurate prediction of PPI $ \Delta \Delta G $ usually requires the availability of the PPI mutant structure, which can be generated using various methods. To satisfy this, MpbPPI was designed to flexibly fit the mutant structures from different sources. In this study, we used three tools to build mutant structures: FoldX, MODELLER and AlphaFold2. FoldX substitutes and optimizes original residue structure based on the given wild-type complex, MODELLER generates the structure through homology modeling and AlphaFold2 can predict the protein structure from scratch. Our research found that when MpbPPI is combined with the three mutation structure generation tools FoldX, MODELLER and AlphaFold2, each combination achieves good prediction results. There is a strong positive correlation between the predicted and measured PPI $ \Delta \Delta G $ values. Specifically, based on the 10-fold cross-validation, for the smallest single mutation dataset, each combination of tools achieved a $ {R}_{\mathrm{P}} $ greater than 0.57. For the multiple-mutation dataset, each combination of tools achieved a $ {R}_{\mathrm{P}} $ more than 0.76. These findings further confirmed MpbPPI is a dependable and efficient method for predicting PPI $ \Delta \Delta G $. Moreover, it can serve as a foundational framework and be flexibly combined with various mutation generation tools to improve the accuracy of PPI $ \Delta \Delta G $ predictions.

**Figure 4:**
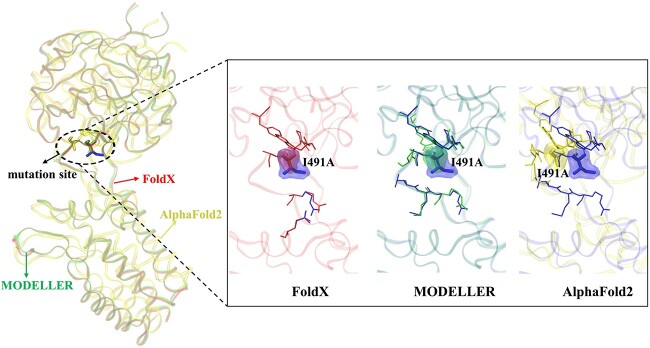
Comparison of mutant PPI structures from various mutant generation tools. An example of the structural differences between WT and mutant structures (PDB ID: 1AK4). The WT structure and mutant structures generated by FoldX, MODELLER and AlphaFold2 are shown in different colours for better identification. The mutant amino acid and its neighboring amino acids’ backbone and side chain are represented as sticks, with the mutant amino acid highlighted in surface style. The Cα RMSD values between the WT and mutant structures were 0 Å for FoldX, 0.3 Å for MODELLER and 3.8 Å for AlphaFold2.

Our results also showed that the FoldX-based MpbPPI prediction outperformed the MODELLER-based prediction, with the AlphaFold2-based prediction yielding the worst results. We observed that, compared with the WT structures, the mutant structures generated by FoldX mainly exhibited conformational changes in the side chain of the mutant residue and their neighboring residues (Figure 4). Meanwhile, MODELLER-based mutant structures showed slight alterations in both the side chain and backbone of the mutant residue and their neighboring residues (Figure 4). AlphaFold2 displayed the largest variations, with the Cα root-mean-square deviation (RMSD) between the WT and AlphaFold2-based mutant structures ranging from 0.7 to 40.6 Å in the M1101 dataset. Among these, the mutation structures generated by AlphaFold2 exhibit larger conformational changes, providing a wider learning space for the model. However, this also increases the prediction complexity, necessitating more data during training to improve the model’s prediction accuracy. Besides, for FoldX and MODELLER, a mutant structure usually can be generated within 1–2 minutes, while AlphaFold2 needs an average of dozens of minutes. Therefore, when considering the conformational changes after protein mutations, striking a balance between the prediction complexity and the amount of training data is needed to achieve better prediction accuracy.

A future direction of our work is to include more flexible biochemical and evolutionary information compatible with existing different-scale geometric characteristics and pre-training tasks. We plan to incorporate the features like the multiple types of PPI interface information, to make our framework able to better adapt to some more specific application scenarios, for example, predicting the PPI $\Delta \Delta G$ in the case that most of the AA mutations occur at the interface of protein–protein complexes.

## METHODS

### Generation of the refined residue-level contact graphs for protein–protein complexes

Before the generation of the residue-level contact graphs, to ensure that each residue node contains complete atom geometry coordinate information, we used FoldX to complete all side chains (based on raw PDB files) in both the pre-training and downstream sets. At the same time, all H atoms were removed from these PDB files.

We then modeled a given PPI complex as a residue contact graph $\mathcal{G}=\left(\mathcal{V},\mathcal{E}\right)$, where $\mathcal{V}$ is the set of residues in the PPI complex (the position of each residue node is determined by its Cα 3D coordinate) and $\mathcal{E}$ is the set of edges between them. The procedure for defining $\mathcal{E}$ is outlined below:

In order to capture more comprehensive interactive relationships for residue nodes in $\mathcal{G}$ (i.e. considering different inter-residue geometric contact relationships with different edge distributions [35]), we defined an independent $\mathrm{K}$-nearest neighbor (KNN) graph ${\mathcal{G}}^{\mathrm{K}}=\left({\mathcal{V}}^{\mathrm{K}},{\mathcal{E}}^{\mathrm{K}}\right)$ (where each node $i$ connects other $\mathrm{K}$ nodes nearest to it, based on the Euclidean distance between node position coordinates) and a radius graph ${\mathcal{G}}^{\mathrm{R}}=\left({\mathcal{V}}^{\mathrm{R}},{\mathcal{E}}^{\mathrm{R}}\right)$ (where each node $i$ connects all other nodes within a $\mathrm{R}$-radius 3D sphere centered at $i$) for each protein–protein complex. For each ${\mathcal{G}}^{\mathrm{K}}$ and ${\mathcal{G}}^{\mathrm{R}}$, $\mathrm{K}$ and $\mathrm{R}$ are set to 20 and 10 Å, and every residue node $i$ in both ${\mathcal{G}}^{\mathrm{K}}$ and ${\mathcal{G}}^{\mathrm{R}}$ is equipped with two coordinate sets ${\mathrm{C}}_i^B$ and ${\mathrm{C}}_i^S$ containing the backbone atom (N, Cα, C, O) coordinates and side chain atom coordinates (removing H), respectively (for a given residue, in both KNN and radius graphs, the contained residue nodes and initial ${\mathrm{C}}_i^B$ and ${\mathrm{C}}_i^S$ are the same).

#### Graph node features

In order to generate (initial) residue node and edge features ${f}_i$ and ${f}_{\left(i,j\right)}$ for $\mathcal{G}$, we first calculated the centroid of $\mathcal{G}$ based on its all raw backbone atom (N, Cα, C, O) coordinates, and preliminarily normalized all the backbone and side chain coordinate sets by subtracting the calculated centroid coordinate value (to get ${\mathrm{C}}^{B^{\prime }}$ and ${\mathrm{C}}^{S^{\prime }}$, representing all backbone and side chain atom sets in current $\mathcal{G}$). Next, in order to fully characterize the geometric relationships between side chain atoms and between side chain and residue backbone atoms within the residue $i$ from different perspectives, node features ${f}_i$ were defined by concatenating the following seven feature sets:

- The centroid of side chain atoms (vector)
- The center of mass of side chain atoms (vector)
- The maximum coordinate value of side chain atoms (vector)
- The unit vector of Cα$-$side chain geometric relationships (vector)
- The normalized Cα coordinate (vector)
- Solvent-accessible surface area (SASA) (scalar)
- Residue interface information (scalar)

The detailed calculation description and meaning of these features are provided in Supplementary Material.

Other than the above seven types of residue node features, we also added five additional node features for providing AA sequence information and close neighboring relationships of a residue, in which ${\mathrm{C}\mathrm{\alpha}}_i^{\prime }$ and ${\mathrm{C}\mathrm{\beta}}_i^{\prime }$ represent the Cα and $\mathrm{C}\mathrm{\beta}$ coordinate of residue $i$ after the preliminary centroid-based normalization:

- The amino acid (AA) type, given as a one-hot representation (scalar)
- Chain index (scalar)
- Sine–cosine encoded dihedral angles (scalar)
- Unit vector encoding of the ${\mathrm{C}\mathrm{\alpha}}_i^{\prime }$ close neighbors (vector)
- Unit vector encoding of the ${\mathrm{C}\mathrm{\alpha}}_i^{\prime }-{\mathrm{C}\mathrm{\beta}}_i^{\prime }$ orientation (vector)

#### Graph edge features

To create edge features ${f}_{\left(i,j\right)}$ capturing interactive contact relationships between residue nodes in current $\mathcal{G}$ [here denoting edges from nodes $j$ to $i$ as $\left(i,j\right)$], we concatenated the following feature sets:

- The unit vector in the direction of $ {\mathrm{C}\mathrm{\alpha}}_j^{\prime }-{\mathrm{C}\mathrm{\alpha}}_i^{\prime } $ (vector)
- Euclidean distance encoding between ${\mathrm{C}\mathrm{\alpha}}_j^{\prime }\ \mathrm{and}\ {\mathrm{C}\mathrm{\alpha}}_i^{\prime }$ based on the Gaussian radial basis functions (scalar)
- Sine–cosine encoded $j-i$ node index (scalar).

For the sine–cosine and Gaussian radial basis encoding methods, we suggest referring Jing et al. for the detailed description [36].

For the scalar and vector features of a residue node $i$, we concatenated them separately, to produce the node scalar features ${f}_i^S$ ($R\in \mathcal{V}\times 29$), node vector features ${f}_i^V$ ($R\in \mathcal{V}\times 9\times 3$), edge scalar features ${f}_{\left(i,j\right)}^S$ ($R\in \mathcal{E}\times 32$, from residue node $j$ to $i$) and edge vector features ${f}_{\left(i,j\right)}^V$ ($R\in \mathcal{E}\times 1\times 3$, from residue node $j$ to $i$), which will be encoded by our GEE layers (as the input) independently. Please note that due to the different generation principles of geometric interactive edges, the edge features of residue $i$ in the KNN and radius contact graphs are different (while the initial node features are exactly the same in the two graphs).

### The GEE encoder and its pre-training techniques

The equivariance property is of importance in protein-related representation learning based on machine learning. Specifically, for a protein structure, its learned representation should not be sensitive to the imposition of protein’s rigid motions (e.g. extra translation to original protein 3D coordinates) because the physical law controlling the dynamics of molecules is not influenced by such motions [37–39]. In order to incorporate such a property into our framework flexibly, we considered employing an equivariant-based graph neural network (GNN) backbone GVP-GNN [36], which uses a message passing strategy [40] for geometric neighbor-based learning of (residue) node-level representations. However, GVP-GNN cannot effectively process the heterogeneous graphs in which nodes or edges could have multiple types, and so unable to satisfy our requirement of performing the KNN and radius graph learning simultaneously (the original equations of GVP-GNN are provided in Supplementary Material). To solve this problem, we extended GVP-GNN into a multi-relational mode as follows to create our geometric equivariant encoder (GEE) layer (in which the superscripts $KNN$ and $radius$ represent the feature/message specific to corresponding contact graphs separately):


(1) \begin{equation*} {f}_{\left(i,j\right)}^{KNN\_ message}=\mathrm{GVP}\left(\mathrm{concat}\left({f}_j^{\left(S,V\right)},{f}_{\left(i,j\right)}^{KNN\left(S,V\right)}\right)\right) \end{equation*}

(2) \begin{equation*} {f}_{\left(i,j\right)}^{radius\_ message}=\mathrm{GVP}\left(\mathrm{concat}\left({f}_j^{\left(S,V\right)},{f}_{\left(i,j\right)}^{radius\left(S,V\right)}\right)\right) \end{equation*}

(3) \begin{equation*} {f}_i^{KNN\_ aggregation}=\mathrm{mean}\left(\sum_{j: edges\ of\ j\ to\ i\ \epsilon\ \mathcal{E}\ in\ KNN\ graph}{f}_{\left(i,j\right)}^{KNN\_ message}\right) \end{equation*}

(4) \begin{equation*} {f}_i^{radius\_ aggregation}=\mathrm{mean}\left(\sum_{j: edges\ of\ j\ to\ i\ \epsilon\ \mathcal{E}\ in\ radius\ graph}{f}_{\left(i,j\right)}^{radius\_ message}\right) \end{equation*}

(5) \begin{eqnarray*} && {f}_i^{\left(S,V\right)}=\mathrm{LayerNorm}\left({f}_i^{\left(S,V\right)}+\mathrm{Dropout}\left(\mathrm{concat}\left({f}_{\left(i,j\right)}^{KNN\_ aggregation},\right.\right.\right. \nonumber \\ && \left.\left.\left. \qquad\qquad{f}_{\left(i,j\right)}^{radius\_ aggregation}\right)\right)\right) \end{eqnarray*}

(6) \begin{equation*} {f}_i^{\left(S,V\right)}=\mathrm{Layernorm}\left({f}_i^{\left(S,V\right)}+\mathrm{Dropout}\left(\mathrm{GVP}\left({f}_i^{\left(S,V\right)}\right)\right)\right) \end{equation*}

Here, we adopted the notation ${f}_j^{\left(S,V\right)}$ and ${f}_{\left(i,j\right)}^{\left(S,V\right)}$ to refer to the tuples of ${f}_j^S$ and ${f}_j^V$ and the tuples of ${f}_{\left(i,j\right)}^S$ and ${f}_{\left(i,j\right)}^V$, respectively. GVP is a special neural network module which is mathematically demonstrated to be insensitive to 3D rigid motions [36]. ${f}_{\left(i,j\right)}^{message}$ represents the message representation flowing/aggregating from residue node $j$ to $i$ during message passing. Intuitively, the message passing of geometric characteristics with different scales in different PPI contact graphs could generate enough powerful representation for the downstream PPI $\Delta \Delta G$ predictions. In addition, we stacked five GEE layers [i.e. Equations (1)–(6)] to constitute the GEE encoder to increase its ability to non-linear fitting, and the hidden feature dimensions for ${f}_j^S$, ${f}_j^V$, ${f}_{\left(i,j\right)}^S$, ${f}_{\left(i,j\right)}^V$ in each layer were the same i.e. 256, 16, 32, 1, respectively (Figure 2A and B).

Based on the above GEE encoder and the designed contact graphs for each protein–protein complex in our strictly screened pre-training set, our multi-task pre-training strategy was devised, in which the backbone denoising, side chain denoising, SASA prediction and AA type prediction tasks were included, which will be performed simultaneously. Intuitively, setting multiple pre-training tasks, which share closely related geometric information of the residues themselves, could be beneficial to capturing comprehensive interactive contact relationships (regulations) within a protein–protein complex. Due to the page limitation, please see the detailed description and meaning of these tasks in Supplementary Material. Concisely, for each protein–protein complex in the pre-training set, we first randomly selected 15% of its residues and set a (boolean) mask to these residues. For these masked residues, we added noise to their backbone and side chain atom coordinates to corrupt the complex’s overall conformation, and then the corrupted conformation (information) was used to recover the complex’s corresponding ground truth conformation or properties (based on the task-specific multi-layer perceptions).

Besides, according to the ground truth to be recovered, the loss functions measuring the model reconstruction errors were mean square error MSE (for backbone denoising, ${MSE}^{C\alpha}$), MSE (for side chain denoising, ${MSE}^{Sidec}$), MSE (for SASA prediction, ${MSE}^{SASA}$) and binary cross-entropy BCE (for AA type prediction, ${BCE}^{AA}$), respectively. To summarize, we can perform the above four pre-training tasks simultaneously in an end-to-end fashion (Figure 2A). The overall learning objective for guiding the model optimization is formulated as follows, and the Adam [41] with the initial learning rate of 0.001 is adopted as the corresponding optimizer:


(9) \begin{equation*} {\ell}^{overall}={MSE}^{C\alpha}+{MSE}^{Sidec}+{MSE}^{SASA}+{BCE}^{AA} \end{equation*}

Furthermore, the choice of the masked residues and magnitude of noise (the details of noise injection is in Supplementary Material) is selected ‘on-the-fly’. In other words, for every epoch in the pre-training, both the masked residues and magnitude of added noise will be determined again, in order to allow the model to see complexes with more diversities during training. The numbers of epochs and early stopping steps were set to 100 and 30, respectively.

For the implementation of the GEE encoder, Pytorch [42] with a default random seed 1234 was employed, and it was trained on a configuration of one NVIDIA A100 GPU.

### The prediction of downstream PPI $\Delta \Delta G$

After pre-training, the GEE encoder has learned how to provide an effective structural representation of a protein–protein complex with good generalization, which can be used for the downstream $\Delta \Delta G$ prediction task (Figure 2B). Specifically, for one PPI AA mutation sample point, we first constructed its KNN and radius contact graphs for the WT and MT 3D complex structures (produced by molecular generation tools), respectively. After that, the interface residues under the WT status were found based on the interfaceResidue function in PyMOL [43] (detailed in Supplementary Material), denoted as ${S}^{interface}$, and we marked current mutation residues as ${S}^{mutation}$. Next, the contact graphs of the WT and MT complexes are sent to the trained GEE encoder, to acquire two groups of residue representations for the whole WT (denoted as ${R}^{WT}$) and MT (denoted as ${R}^{MT}$) structures, respectively.

After acquiring ${R}^{WT}$ and ${R}^{MT}$, following Liu et al. [15], we leveraged gradient-boosting trees (GBT) as the final $\Delta \Delta G$ decoder/predictor due to its good capability to effectively handle high-dimensional features and overcome overfitting under relatively small datasets. To get the informative input of GBT, we generated the following representations for comparing the geometric difference between WT and MT structures from different perspectives:

- Max-pooling and mean-pooling of representations of mutation sites in WT complex: $\max \_\mathrm{pooling}\left({R}^{WT}\epsilon\ {S}^{mutation}\right)$ and $\mathrm{mean}\_\mathrm{pooling}\left({R}^{WT}\epsilon\ {S}^{mutation}\right)$.
- Max-pooling and mean-pooling of representations of interface sites in WT complex: $\max \_\mathrm{pooling}\left({R}^{WT}\epsilon\ {S}^{interface}\right)$ and $\mathrm{mean}\_\mathrm{pooling}\left({R}^{WT}\epsilon\ {S}^{interface}\right)$.
- Max-pooling and mean-pooling of representations of mutation sites in MT complex: $\max \_\mathrm{pooling}\left({R}^{MT}\epsilon\ {S}^{mutation}\right)$ and $\mathrm{mean}\_\mathrm{pooling}\left({R}^{MT}\epsilon\ {S}^{mutation}\right)$.
- Max-pooling and mean-pooling of representations of interface sites in MT complex: $\max \_\mathrm{pooling}\left({R}^{MT}\epsilon\ {S}^{interface}\right)$ and $\mathrm{mean}\_\mathrm{pooling}\left({R}^{MT}\epsilon\ {S}^{interface}\right)$.
- The difference between the mutation sites in WT and MT complexes: $\max \_\mathrm{pooling}\left({R}^{WT}\epsilon\ {S}^{mutation}\right)-\max \_\mathrm{pooling} \left({R}^{MT}\epsilon\ {S}^{mutation}\right)$ and $\mathrm{mean}\_\mathrm{pooling}\left({R}^{WT}\epsilon\ {S}^{mutation}\right)-\mathrm{mean}\_\mathrm{pooling}\left({R}^{MT}\epsilon\ {S}^{mutation}\right)$.
- The global geometric representation of MT complex: $\mathrm{mean}\_\mathrm{pooling}\left({R}^{MT}\right)$.

Above representations will be concatenated together and sent to the GBT decoder, and the output of GBT is the final predicted PPI $\Delta \Delta G$ representing the binding affinity/binding free energy change from wild-type to mutant status (i.e. $\Delta{G}^{WT}-\Delta{G}^{MT}$). In addition, for the selection of GBT hyper-parameters, the learning rate, number of sub-estimators and maximum depth of sub-estimators are set to 0.001, 5 × 10^4^ and 6, respectively. The implementation of this GBT was based on the scikit-learn library [44]. An overall summary of tools for implementing MpbPPI is provided in Supplementary Material.

**Key Points**

- We introduce MpbPPI, a flexible geometric equivariant graph neural network framework, which effectively propagates specially designed different-scale geometric relationship characteristics of both wild-type and mutant protein–protein complexes, providing high-quality low-dimensional representations for protein–protein interaction (PPI) $\Delta \Delta G$ predictions caused by amino acid site mutations.
- To facilitate MpbPPI learning multi-scale geometric regulations of protein–protein complexes, we devise four pre-training tasks aiming at learning the geometric properties of individual residues themselves from different perspectives, for forcing MpbPPI to capture the comprehensive geometric regulations based on a strictly screened protein complex pre-training dataset.
- MpbPPI can flexibly accept the mutant structures from various generation tools (e.g. FoldX, MODELLER, AlphaFold2) as the model input, allowing the evaluation and comparison of different generation tools to choose the most appropriate method for a given PPI system.
- The effectiveness of MpbPPI on predicting PPI $\Delta \Delta G$ has been extensively validated on four benchmark datasets under two realistic evaluation settings.

## Supplementary Material

Supplementary_Material_bbad310

## Data Availability

The MpbPPI models and the used data can be found in https://github.com/arantir123/MpbPPI.

## Author Biographies

**Yang Yue** is a PhD student of the School of Computer Science from the University of Birmingham, UK. His research interests include bioinformatics, machine learning and data mining.

**Shu Li** is a lecturer in the Centre for Artificial Intelligence Driven Drug Discovery at Macao Polytechnic University. Her research interests are computer-aided molecular design and hybrid force field development.

**Lingling Wang** is a PhD student in the Centre for Artificial Intelligence Driven Drug Discovery at Macao Polytechnic University. Her research interests are computer-aided drug design and molecular modeling.

**Huanxiang Liu** is a professor in the Centre for Artificial Intelligence Driven Drug Discovery at Macao Polytechnic University. Her research interests are computer-aided molecular design and molecular modeling of protein structure and function.

**Henry H.Y. Tong** is a professor in the Centre for Artificial Intelligence Driven Drug Discovery at Macao Polytechnic University. His research interests are pharmaceutics and dosage form science.

**Shan He** is an associate professor at the School of Computer Science, the University of Birmingham. His research interests are machine learning, evolutionary algorithms, network medicine and drug discovery.
