# Supplementary material for: MpbPPI: a multi-task pre-training-based equivariant approach for the prediction of the effect of amino acid mutations on protein–protein interactions
Source: Brief Bioinform. 2023 Aug 31;24(5):bbad310. doi: 10.1093/bib/bbad310 (PMC10516393; doi:10.1093/bib/bbad310)
Supplement: Supplementary_Material_bbad310 [file supplementary_material_bbad310.docx]

**Supplementary Information (SI)**

**MpbPPI: a multi-task pre-training-based equivariant approach for the prediction of the effect of amino acid mutations on protein-protein interactions**

Yang Yue, Shu Li, Lingling Wang, Huanxiang Liu, Henry H. Y. Tong, Shan He^*^

Yang Yue and Shu Li contributed equally to this work. Corresponding author: Shan He, Centre for Computational Biology, School of Computer Science, The University of Birmingham, Edgbaston, Birmingham, B15 2TT, UK. Tel: 44-1214142775; Fax: 44-1214144281; E-mail: s.he@cs.bham.ac.uk

**Yang Yue** is a PhD student of the School of Computer Science from the University of Birmingham, UK. His research interests include bioinformatics, machine learning and data mining.

**Shu Li** is a lecturer in Centre for Artificial Intelligence Driven Drug Discovery at Macao Polytechnic University. Her research interests are computer-aided molecular design and hybrid force field development.

**Lingling Wang** is a PhD student in Centre for Artificial Intelligence Driven Drug Discovery at Macao Polytechnic University. Her research interests are computer-aided drug design and molecular modeling.

**Huanxiang Liu** is a professor in Centre for Artificial Intelligence Driven Drug Discovery at Macao Polytechnic University. Her research interests are computer-aided molecular design and molecular modeling of protein structure and function.

**Henry H. Y. Tong** is a professor in Centre for Artificial Intelligence Driven Drug Discovery at Macao Polytechnic University. His research interests are pharmaceutics and dosage form science.

**Shan He** is an associate professor at the School of Computer Science, the University of Birmingham. His research interests are machine learning, evolutionary algorithms, network medicine and drug discovery.

**Pre-training dataset collection procedure**

To retrieve enough real protein-protein complexes for the pre-training, we first collected all raw protein-protein complex Protein Data Bank (PDB) files from the PDBbind (PDBbind v2020 protein-protein complexes)^1^ and 3DComplex (QS40 level)^2^ databases. In order to prevent implicit information leakage, based on their PDB ID, we removed the complexes which share either high topological structural similarity or sequence identity with the complexes in downstream datasets from above two datasets. This is done by 1) ignoring the complexes included in all downstream datasets, 2) ignoring incomplete complexes (i.e., the complexes with only Cα in backbone atoms) and over-large complexes (i.e., the complexes with residue number over 3000), and 3) performing further complex screening based on the Evolutionary Classification of protein Domains (ECOD) classification system^3^.

For the ECOD-based complex screening, specifically, ECOD has clustered a large number of commonly-seen protein-protein complexes based on their protein domains under different pre-defined scales, the complexes clustered into the same group (under a certain pre-defined scale) represent that their included domains share high similarity under the current scale. For example, domains in the same T-scale group are the domains sharing similar topological connections, F-group groups domains with significant sequence similarity in a family. Based on this, we first downloaded the off-line ECOD classification file (ecod.develop287.domains.txt), and retrieved the T-group and F-group category sets based on protein domains included in every wide-type protein-protein complex of all downstream datasets. Next, for any complex in PDBbind or 3DComplex, it was selected as a pre-training sample only if there was no overlap between the allocated effective T-group/F-group categories of its any domains and the above T-group/F-group category sets. After that, 14936 complexes were collected in total, all of which share low topological and sequence similarity with all downstream data.

**The generation of the mutant protein-protein complex structure**

In the downstream $\Delta\Delta G$ prediction task, we considered three types of mainstream molecular generation tools for providing mutant (MT) protein-protein complex structures for performance comparison, including FoldX, MODELLER, and AlphaFold2.

Specifically, for FoldX, we used the BuildModel function in FoldX 4.0, inputting the wild-type (WT) structure and corresponding mutation information (mutation sites and AA mutation types), to produce the mutant structure.

For MODELLER, the structure for a single mutation or multiple point mutations was modeled based on its WT structure using homologous modeling with MODELLER 10.4. We added chain break (‘/’) characters to the alignment file. Then we used the AutoModel class to build models of the complex.

For AlphaFold2, we used a locally installed version of the publicly available ColabFold^4^ to predict mutant structures. The amino acid sequence was modified to reflect the desired mutations, and this updated sequence was used as input for the AlphaFold2. Following the AlphaFold2 pipeline, we performed a MSA search with MMseqs2 on the UniRef30 database^5^ and generated five models for each sample. These models were subsequently relaxed with the Amber program and ranked. We ultimately selected the top-ranked structure for $\Delta\Delta G$ prediction tasks.

**Baseline Methods**

We compare our MpbPPI against seven baseline methods in total, including DGCddG, PerSpect-E, GeoPPI, TopNetTree, FoldX, Flex ddG, and MM/GBSA. We briefly describe these methods as follows.

1. DGCddG^6^: DGCddG is a graph convolutional network (GCN)-based method, mainly designed for estimating PPI $\Delta\Delta G$ caused by amino acid (AA) mutations occurring at PPI interfaces. It creates residue-level contact graphs for WT and MT protein-protein complexes, in which edges in the WT graph are constructed based on the distance threshold between residues and the mutant graph is created by simply replacing WT AA nodes to MT AA nodes. Besides, it uses biochemical (e.g., polarity) and evolution information (e.g., PSI-Blast profile) as node features. The final PPI $\Delta\Delta G$ is predicted based on the WT and MT representations from a multi-layer GCN and extra DSSP^7^ information.
2. PerSpect-EL^8^: PerSpect-EL is designed to use persistent spectral-based features that capture molecular-level information of PPI systems represented by a series of simplicial complexes. The predictions are made by the stack of three different types of base learners.
3. GeoPPI^9^: GeoPPI is a GCN-based method for capturing atom-level relationships, different from DGCddG, its construction of WT and MT complex graphs is based on atom distance relationships. Specifically, GeoPPI creates atom-level graphs for the WT and MT structures, in which an edge is added if the distance between two atoms is smaller than 3$Å$ and an extra cutoff is set to only retain atoms around mutation or interface sites to save the computational cost. To better capture the atom-level relationships in complex graphs, a single-task perturbation-based pre-training task is utilized to train the GCN model.
4. TopNetTree^10^: TopNetTree aims to capture inter-atom geometric relationships of protein-protein complexes based on an algebraic topology techniques named persistent homology^11^, to generate the representation for the protein-protein complex considering mutation information. Furthermore, the generated PPI representation is further enhanced by a convolutional neural network (CNN) and sent to a gradient-boosting trees (GBT) model for PPI $\Delta\Delta G$ prediction.
5. FoldX^12^: FoldX is an empirical energy-based tool, which contains the function aiming at using protein force-fields as the proxy to simulate PPI $\Delta\Delta G$ caused by AA mutations.
6. Flex ddG^13^: Rosetta Flex ddG is the latest protein affinity maturation module released in 2018. It uses "backrub" to create a set of models after sampling the conformation, minimizing torsion, repacking the side chain, and average to the set method to predict the interface binding affinity.
7. MM/GBSA^14,15^: Molecular Mechanics Generalized Born Surface Area (MM/GBSA) is a free energy prediction method developed by Kollman et al. and balances the computational accuracy and efficiency between the docking score function method and the alchemical free energy method. In this study, the proteins were originally mutated using PyMOL^16^. The protein-protein complex systems were constructed using the “tleap” program in Amber22, and the AMBER ff14SB force field was optimized for the protein. In order to avoid unexpected local maxima, 500 steps of minimizations were carried out for each protein-protein complex using the steepest descent and conjugate gradient method, where the cutoff point for non-bonded interactions was set to 12 Å. The “sander” program in Amber22 was utilized to carry out the minimizations. We perform MM/GBSA calculations based on the results of the energy minimization of the complex system using the MMPBSA.py module in AMBER22.

**Experiment about correlating sequence representation difference to** **the PPI** $\boldsymbol{\Delta\Delta G}$ **value**

To investigate the results of directly correlating difference (e.g. distance, similarity) between sequence representations of the mutant and wildtype complexes, to the PPI $\Delta\Delta G$ value, we did an experiment as follows:

Under the largest dataset S4169, for each sample point, we first retrieved all initial residue node features corresponding to the WT and mutant protein-protein complexes separately (i.e., these are the residue features before being sent to the trained GEE encoder and GBT-based decoder). Then, to generate the (sequence) representation of the WT sequence, we concatenated the corresponding residue node features following the order of the WT residue sequence. As an analogy, the representation of the mutant sequence can also be generated (these two representations have the same length for the same sample point). Next, we calculated the cosine similarity (which are commonly used to compare the difference of representations) between the mutant and WT sequence representation for each sample point, and used Pearson’s correlation coefficient ($R_{P}$) to correlate the calculated cosine similarity to the PPI $\Delta\Delta G$ ground truth. The $R_{P}$ on S4169 was 0.255 while our tenfold cross-validation result was 0.795 (based on FoldX), which further demonstrated the necessity of training a predictive model on top of these representations.

**Experiment about reproducing results of some representative baseline methods based on available information**

We re-produced the results of GeoPPI on the S4169 dataset and TopNetTree on the S645 and S1131 datasets. Specifically, GeoPPI is a representative geometric property pre-training-based method and TopNetTree is also an important topology-based machine learning method for PPI $\Delta\Delta G$ predictions. Furthermore, GeoPPI provides the pre-trained feature encoder/embedder and exact hyper-parameters of its decoder on the S4169 dataset and TopNetTree provides the already pre-processed sample point features (and corresponding decoder hyper-parameters) on the S645 and S1131 datasets, on which we can apply the identical tenfold cross-validation settings for comparing them (Supplementary Table 1).

Supplementary Table 1: $R_{P}$ results of the re-produced representative methods

| **Name** | **Dataset** | $\boldsymbol{R}_{\mathbf{P}}$ |
| --- | --- | --- |
| TopNetTree | S645 | 0.648±0.007 |
| TopNetTree | S1131 | 0.857±0.003 |
| GeoPPI | S4169 | 0.719±0.006 |

From the results, we observed that, MpbPPI still outperformed TopNetTree and GeoPPI on relatively larger datasets S1131 (MpbPPI-FoldX: 0.865±0.003) and S4169 (MpbPPI-FoldX: 0.795±0.004), respectively (except for TopNetTree on S645 (MpbPPI-FoldX: 0.615±0.013)), which further demonstrated the effectiveness of our proposed framework.

**Detailed description of the protein-protein complex type-based data splitting**

The specific data splitting pipeline can be summarized as follows:

1. For a downstream PPI $\Delta\Delta G$ dataset, we first obtain its included WT protein-protein complex types, and count the sample point number for each complex type. Then, sort the complex types based on their corresponding sample point numbers from large to small.
2. For the sorted WT complex types, we iterate all of them (from large to small based on their sample point numbers) and put every five in one bin, and the last bin could be incomplete as the total number of WT complex types may not be a multiple of 5.
3. For all bins except for the last one, the WT complex types within a bin are randomly shuffled and then are allocated into different five folds (one WT complex type only to one fold). After that, for each fold, the sample points corresponding to its allocated WT complex types are added into this fold. Next, the sample point number contained in each fold is counted.
4. For the last bin, its WT complex types (and the corresponding sample points) are [preferentially](javascript:;) allocated into folds that have fewer existing sample points (one WT complex type only to one fold), which makes the sample point number and WT protein-protein complex type number assigned to each fold to be close.

**The illustrative example of the predicted outcomes of MpbPPI**

The H-Ras/Raf complex is an important component of the cellular signaling pathway, known as the Ras/Raf/MEK/ERK pathway (also called the mitogen-activated protein kinase (MAPK) pathway). This pathway plays a critical role in regulating cell growth, differentiation, survival, and apoptosis. MpbPPI predicted $\Delta\Delta G$ values of 1.44 kcal/mol and 1.61 kcal/mol for H-Ras protein mutations T35A and E37G in the H-Ras/Raf complex, respectively. These predictions suggested that the two mutations decreased the interaction between H-Ras and Raf, which was consistent with previous experimental finding that these mutations hinder the interaction between H-Ras and Raf^17^. Moreover, antibodies are proteins employed by the immune system to identify and neutralize foreign particles, including viruses and bacteria. Gaining insights into how mutations influence protein-protein interactions can help researchers develop antibodies that exhibit enhanced binding to target proteins. Our model, MpbPPI, has the potential to aid researchers in creating superior antibodies (e.g., help to evaluate $\Delta\Delta G$ between newly designed antibodies with AA alterations and target proteins compared with the original protein-protein interaction systems), ultimately resulting in improved therapeutic approaches and better outcomes for patients.

**The discussion of some optional features for** $\boldsymbol{\Delta\Delta G}$ **predictions**

1. Electrostatic energy information: The interaction between protein complexes is mainly non-bonded interactions, particularly electrostatic interactions. In some previous machine learning or deep learning prediction models, the PPI contact interface electrostatic energy was used as a feature to more accurately predict the impact of amino acid mutations. Electrostatic energy is the energy resulting from the interaction between charges and can be obtained by calculating the Coulomb interaction energy between molecules in the molecular force field. In this study, however, our features only included the sequence and geometric characteristics of the protein structure, without considering energy features related to electrostatic interactions. Notably, the mutant structures input into our model were obtained using FoldX, MODELLER, and AlphaFold, all of which perform energy optimization to obtain the structures. The molecular energy optimization process includes the calculation of electrostatic interaction energy. Therefore, although our features did not explicitly include electrostatic energy, electrostatic interactions may implicitly affect the accuracy of the model through the generated mutated structures.

On top of this, we also discussed the prediction results of charged residues mutations of MpbPPI here: The four test datasets in this study all contain charged amino acids. Specifically, in the largest dataset, S4169, the MpbPPI-FoldX predicted $R_{P}$ values for charged-to-neutral and neutral-to-charged residue mutations were 0.807 and 0.861 (based on the tenfold cross-validation), respectively, which were higher than the overall $R_{P}$ value (0.795) for the dataset. This indicated that our model had good performance in predicting charged residue mutations.

1. Entropic effect information: In PPI, entropy change is often associated with the free energy change that occurs when proteins transition from their unbound state to the bound state^18^. As proteins bind to one another, they generally undergo conformational changes, leading to a decrease in conformational entropy. However, calculating entropy is complex and computationally expensive, posing challenges for incorporating it into both classical computational models (such as molecular dynamics simulations) and machine learning models.

Standard methods for estimating the entropic component include normal mode analysis (NMA)^19^ and the quasi-harmonic approximation^20^. In machine learning, entropic effects may be implicitly incorporated into the feature vector using descriptors that capture the protein's flexibility or mobility, such as the number of rotatable bonds or the radius of gyration^21^.

In this study, we did not create feature vectors that explicitly account for entropy contributions. When evaluating the model's accuracy, it is crucial to consider whether entropy effects are expected to play a significant role in the systems being studied. We assessed the impact of AA mutations in the complex by predicting the relative binding free energy ($\Delta\Delta G$) between the mutant and WT structures. Since the structures before and after the mutation are relatively similar, their contributions to conformational entropy are also similar and can largely cancel each other out. This allows us to disregard the impact of the entropy term.

1. Multiple sequences alignment (MSA) information: Regarding MpbPPI and MSA, MpbPPI did not directly use MSA as input. Instead, it used simple geometric features of the protein complex structure, such as the unit vector of Cα-side chain, to efficiently capture the relationships between the backbone and side chains of the protein. Using these geometric features, MpbPPI was able to predict the effect of AA mutations on protein-protein interactions. However, in the combination of MpbPPI and AlphaFold2, the MSA was used by AlphaFold2 to identify conserved regions and patterns in the protein sequence, which could help guide the prediction of the mutant structure.

In some cases, MSA can provide valuable information about the evolutionary relationships between related proteins and help identify functionally important residues and regions in protein sequences. However, there are some limitations about using MSA as input features, such as the potential for incomplete or biased MSA and the computational expense of generating them. Despite these challenges, MSA remains an effective tool for predicting protein-protein interactions, and many models continue to use them as input features. In the future work, we will attempt to incorporate more flexible biochemical and evolutionary information, which is compatible with the existing geometric features and pre-training tasks under current downstream task, into our MpbPPI framework.

**Detailed description of the residue node feature calculation**

We first denote all the preliminarily normalized backbone and side chain coordinate sets in current contact graph $\mathcal{G}$ (see **Generation of the refined residue-level contact graphs for protein-protein complexes** section of the manuscript), which represent all backbone and side chain atom sets in $\mathcal{G}$, as $C^{B^{'}}$ and $C^{S^{'}}$ (the corresponding sets for an individual residue $i$ are denoted as $C_{i}^{B}$ and $C_{i}^{S}$, respectively). Based on this, the detailed description of devised seven types of node features $f_{i}$ (mentioned in the above section of the manuscript) are as follows, in which the reason for the further normalization is to make all features basically stay in the same scale for the better model optimization:

1. The centroid of side chain atoms (vector): further normalize $C^{B^{'}}$ and $C^{S^{'}}$ based on the largest Euclidean distance between $C^{B^{'}}$ and the origin of coordinate system (i.e., (0, 0, 0)), to acquire $C^{B^{''}}$ and $C^{S^{''}}$, and then the centroid of $C_{i}^{S^{''}}$ is calculated as the residue feature.
2. The center of mass of side chain atoms (vector): based on the calculated $C_{i}^{S^{''}}$, to calculate the center of mass of $C_{i}^{S^{''}}$ as the residue feature (i.e., the centroid weighted by corresponding atom mass).
3. The maximum coordinate value of side chain atoms (vector): based on the above $C_{i}^{S^{''}}$, to retrieve the maximum coordinate values of $C_{i}^{S^{''}}$ on $X-$, $Y-$, $Z-$ axis, respectively.
4. The unit vector of Cα$-$side chain geometric relationships (vector): denote the Cα coordinate of residue $i$ after preliminary centroid-based normalization as ${C\alpha}_{i}^{'}$, calculate the forward and reverse unit vectors in the directions of ${C\alpha}_{i}^{'}-C_{i}^{S^{'}}$ and $C_{i}^{S^{'}}-{C\alpha}_{i}^{'}$ as the residue feature.
5. The normalized Cα coordinate (vector): the Cα coordinate in $C_{i}^{B^{''}}$.
6. Solvent-accessible surface area (SASA) (scalar): calculate the SASA of residue $i$ in current protein-protein complex based on the probe radius 1.4$Å$ (denoted as $\mathrm{SASA}_{complex\_i}$). Such calculated SASA can depict the surface outline of the side chain atoms of a residue, and also suggest the geometric relationships of these atoms^22^.
7. Residue interface information (scalar): PPI interface information has been demonstrated to be effective in PPI $\Delta\Delta G$ predictions^9^. Therefore, based on above $\mathrm{SASA}_{complex\_i}$, further calculate the SASA of residue $i$ in current chain (denoted as $\mathrm{SASA}_{chain\_i}$), if $\mathrm{SASA}_{chain\_i}-\mathrm{SASA}_{complex\_i}$ (i.e., $\mathrm{dASA}_{i}$) is larger than 1 $Å^{2}$, treat $i$ as an interface residue and mark this feature as 1, otherwise mark it as 0.

**Equations of the GVP-GNN backbone**

Concisely, GVP-GNN uses the message passing strategy^23^ for geometric neighboring learning to produce the (residue) node-level representations:

| $f_{(i, j)}^{message}=GVP(concat(f_{j}^{\left( S, V \right)},f_{(i,j)}^{\left( S, V \right)}))$ | (1) |
| --- | --- |
| $f_{i}^{\left( S, V \right)}=\mathrm{LayerNorm}(f_{i}^{\left( S, V \right)}+\mathrm{mean}(\mathrm{Dropout}(\sum_{j: edges of j to i \epsilon\mathcal{E}} f_{(i, j)}^{message})))$ | (2) |
| $f_{i}^{\left( S, V \right)}=Layernorm(f_{i}^{\left( S, V \right)}+Dropout(GVP(f_{i}^{\left( S, V \right)})))$ | (3) |

in which $f_{j}^{\left( S, V \right)}$ and $f_{(i,j)}^{\left( S, V \right)}$ are the tuples of $f_{j}^{S}$ (i.e., residue node scalar features) and $f_{j}^{V}$ (i.e., residue node vector features) and the tuples of $f_{(i,j)}^{S}$ (i.e., residue edge scalar features) and $f_{(i,j)}^{V}$ (i.e., residue edge vector features), respectively. GVP is a special neural network module which is mathematically demonstrated to be insensitive to 3D rigid motions^24^, and $f_{(i, j)}^{message}$ represents the message representation flowing/aggregating from residue node $j$ to $i$ during message passing.

**Detailed description of the devised pre-training strategies**

1. Backbone denoising: to make the GEE encoder better capture the geometric relationships between residue backbone atoms and potential backbone conformation change brought by different molecular generation tools, the backbone denoising task was devised. For each protein-protein complex, we first randomly selected 15% of its residues and set a (boolean) mask to these residues, and then added noise randomly drawn from the standard normal distribution with the truncation (the maximum and minimum noise values are 2 and -2 separately) to raw backbone atom coordinates (i.e., N, Cα, C, O) of masked residues. Besides, the noise extraction for coordinate corruption is independent for each atom.

After that, all coordinates in the corrupted protein-protein complex (in the pre-training set) were normalized using its backbone atom centroid. Under the normalized coordinates, the KNN and radius contact graphs are generated, and then relevant node and edge geometric features are constructed based on the corrupted graphs. Furthermore, for the masked residues, their absolute Cα coordinates (i.e., the Cα coordinates before centroid-based normalization) before and after the corruption are retrieved as the denoising objective. Specifically, the final representations of masked residues produced by the GEE encoder are sent to a multi-layer perception (MLP), for predicting the relative distance between the above two sets of Cα coordinates. The loss function measuring the denoising error is the mean square error (MSE, denoted as ${MSE}^{C\alpha}$).

1. Side chain denoising: to effectively capture the geometric relationships between side chain atoms and between backbone and side chain atoms on the basis of above backbone conformation change learning, for each of the aforementioned corrupted residues, we first retrieved its side chain coordinate set after the preliminary centroid normalization (i.e., $C_{i}^{S^{'}}$). Then we randomly selected one of its side chain atom coordinates and added above normal distribution noise to this coordinate. Next, we calculated the centroid of side chain atoms, center of mass of side chain atoms, maximum coordinate value of side chain atoms, and unit vector of Cα$-$side chain geometric relationships (detailed in the last section **Detailed description of the node feature calculation**) for the corrupted residues.

The objective for this task is to recover these side chain-related geometric features in the PPI contact graph before corruption, based on those in the contact graph after corruption as well as other corrupted geometric features. The denoising is performed using an independent MLP predictor, and the loss function is also MSE, denoted as ${MSE}^{Sidec}$.

1. SASA prediction: other than the above four types of features that can capture side chain-related geometric properties, residue SASA values in a protein-protein complex are also able to depict such properties from a high-level perspective. Therefore, the SASA prediction task is incorporated, we first set all SASA values in the masked residues to 0, and same to above, we used the MLP and MSE loss (denoted as ${MSE}^{SASA}$) to formulate this prediction task.
2. AA type prediction: the AA type (representation) carries high-level biophysical properties of a residue, if retain it in the masked residues, the learning objectives of the above three pre-training tasks could be more easily to be achieved. In order to improve the model generalization by increasing the difficulty of the training task, for the corrupted residues, their AA type representations are also masked and to be predicted by another MLP, and the loss function used is the binary cross entropy (BCE) loss (denoted as ${BCE}^{AA}$).

**Details of the interface identification based on PyMOL**

In this study, we used a PyMOL script called InterfaceResidues.py to determine whether an amino acid is located at the interface of protein-protein interactions. This script identifies interface residues between two proteins or chains using the following method. First, it calculates the surface area of each amino acid of the protein complex. Then, it separates the complex into two parts, one for each chain, and calculates the surface area of each amino acid of each chain independently. Finally, it calculates the difference in the surface area of each amino acid in the complex and in each chain. This approach is widely used for identifying interface residues in protein complexes and can provide valuable insights into the molecular basis of protein-protein interactions. Therefore, we did not need to explicitly consider the protein interface orientation when building the protein complex. The script can be found in <http://www.protein.osaka-u.ac.jp/rcsfp/supracryst/suzuki/jpxtal/Katsutani/en/interface.php>.

**Summary of implementation tools for MpbPPI**

Our basic program language is Python 3.9.13, on which Pytorch 1.12.1^25^ with a default random seed 1234 was used to construct the framework of the GEE encoder (and corresponding pre-training strategies). For the GBT-based decoder implementation, we applied PyMOL 2.4.1^16^ to search for the interface residues, and scikit-learn library 1.2.2^26^ was adopted to establish the GBT model. For the mutant structure generation, tools/softwares to be considered includes FoldX 4.0, MODELLER 10.4, and AlphaFold2. Besides, FoldX 4.0 was also used to complete the side chains for raw PDB files in both pre-training and downstream sets.

**Supplementary References**

1 Su M, Yang Q, Du Y, et al. Comparative assessment of scoring functions: the CASF-2016 update[J]. Journal of chemical information and modeling, 2018, 59(2): 895-913.

2 Levy E D, Pereira-Leal J B, Chothia C, et al. 3D complex: a structural classification of protein complexes[J]. PLoS computational biology, 2006, 2(11): e155.

3 Cheng H, Schaeffer R D, Liao Y, et al. ECOD: an evolutionary classification of protein domains[J]. PLoS computational biology, 2014, 10(12): e1003926.

4 Mirdita M, Schütze K, Moriwaki Y, et al. ColabFold: making protein folding accessible to all[J]. Nature methods, 2022, 19(6): 679-682.

5 Mirdita M, Steinegger M, Söding J. MMseqs2 desktop and local web server app for fast, interactive sequence searches[J]. Bioinformatics, 2019, 35(16): 2856-2858.

6 Jiang Y, Quan L, Li K, et al. DGCddG: Deep Graph Convolution for Predicting Protein-Protein Binding Affinity Changes Upon Mutations[J]. IEEE/ACM Transactions on Computational Biology and Bioinformatics, 2023.

7 Kabsch W, Sander C. Dictionary of protein secondary structure: pattern recognition of hydrogen‐bonded and geometrical features[J]. Biopolymers: Original Research on Biomolecules, 1983, 22(12): 2577-2637.

8 Wee J J, Xia K. Persistent spectral based ensemble learning (PerSpect-EL) for protein–protein binding affinity prediction[J]. Briefings in Bioinformatics, 2022, 23(2): bbac024.

9 Liu X, Luo Y, Li P, et al. Deep geometric representations for modeling effects of mutations on protein-protein binding affinity[J]. PLoS computational biology, 2021, 17(8): e1009284.

10 Wang M, Cang Z, Wei G W. A topology-based network tree for the prediction of protein–protein binding affinity changes following mutation[J]. Nature Machine Intelligence, 2020, 2(2): 116-123.

11 Edelsbrunner H, Letscher D, Zomorodian A. Proceedings 41st annual symposium on foundations of computer science[J]. 2000.

12 Schymkowitz J, Borg J, Stricher F, et al. The FoldX web server: an online force field[J]. Nucleic acids research, 2005, 33(suppl_2): W382-W388.

13 Barlow K A, Ó Conchúir S, Thompson S, et al. Flex ddG: Rosetta ensemble-based estimation of changes in protein–protein binding affinity upon mutation[J]. The Journal of Physical Chemistry B, 2018, 122(21): 5389-5399.

14 Wang E, Sun H, Wang J, et al. End-point binding free energy calculation with MM/PBSA and MM/GBSA: strategies and applications in drug design[J]. Chemical reviews, 2019, 119(16): 9478-9508.

15 Salomon‐Ferrer R, Case D A, Walker R C. An overview of the Amber biomolecular simulation package[J]. Wiley Interdisciplinary Reviews: Computational Molecular Science, 2013, 3(2): 198-210.

16 DeLano W L. Pymol: An open-source molecular graphics tool[J]. CCP4 Newsl. Protein Crystallogr, 2002, 40(1): 82-92.

17 Drugan J K, Khosravi-Far R, White M A, et al. Ras Interaction with Two Distinct Binding Domains in Raf-1 5 Be Required for Ras Transformation (∗)[J]. Journal of Biological Chemistry, 1996, 271(1): 233-237.

18 Kamisetty H, Ramanathan A, Bailey‐Kellogg C, et al. Accounting for conformational entropy in predicting binding free energies of protein‐protein interactions[J]. Proteins: Structure, Function, and Bioinformatics, 2011, 79(2): 444-462.

19 Genheden S, Kuhn O, Mikulskis P, et al. The normal-mode entropy in the MM/GBSA method: effect of system truncation, buffer region, and dielectric constant[J]. Journal of chemical information and modeling, 2012, 52(8): 2079-2088.

20 Chang C E, Chen W, Gilson M K. Evaluating the accuracy of the quasiharmonic approximation[J]. Journal of Chemical Theory and Computation, 2005, 1(5): 1017-1028.

21 Rose G D. Reframing the protein folding problem: Entropy as organizer[J]. Biochemistry, 2021, 60(49): 3753-3761.

22 Liu Y, Zhang L, Wang W, et al. Rotamer-free protein sequence design based on deep learning and self-consistency[J]. Nature Computational Science, 2022, 2(7): 451-462.

23 Gilmer J, Schoenholz S S, Riley P F, et al. Neural message passing for quantum chemistry[C]//International conference on machine learning. PMLR, 2017: 1263-1272.

24 Jing B, Eismann S, Suriana P, et al. Learning from protein structure with geometric vector perceptrons[J]. arXiv preprint arXiv:2009.01411, 2020.

25 Paszke A, Gross S, Massa F, et al. Pytorch: An imperative style, high-performance deep learning library[J]. Advances in neural information processing systems, 2019, 32.

26 Pedregosa F, Varoquaux G, Gramfort A, et al. Scikit-learn: Machine learning in Python[J]. the Journal of machine Learning research, 2011, 12: 2825-2830.

Supplementary Table 2: The RMSE and MAE results of MpbPPI under the mutation-level tenfold cross-validation on the four PPI $\Delta\Delta G$ datasets. The results of MpbPPI were reported based on the average of five times of the independent tenfold cross-validation runs.

| **S645** | **RMSE** | **MAE** |
| --- | --- | --- |
| MpbPPI-FoldX | **1.537±0.018** | **0.971±0.010** |
| MpbPPI-MODELLER | 1.562±0.010 | 0.996±0.003 |
| MpbPPI-AlphaFold2 | 1.590±0.022 | 1.018±0.011 |
| **M1101** | **RMSE** | **MAE** |
| MpbPPI-FoldX | **1.317±0.015** | **0.839±0.008** |
| MpbPPI-MODELLER | 1.338±0.013 | 0.861±0.008 |
| MpbPPI-AlphaFold2 | 1.372±0.020 | 0.881±0.008 |
| **S1131** | **RMSE** | **MAE** |
| MpbPPI-FoldX | **1.250±0.004** | **0.878±0.004** |
| MpbPPI-MODELLER | 1.328±0.004 | 0.966±0.003 |
| **S4169** | **RMSE** | **MAE** |
| MpbPPI-FoldX | **1.073±0.004** | **0.717±0.003** |
| MpbPPI-MODELLER | 1.111±0.008 | 0.754±0.004 |

The bold data indicates the best result under current evaluation metric and dataset.

Supplementary Table 3: The RMSE and MAE results of involved MpbPPI variants under the mutation-level tenfold cross-validation. The results of the MpbPPI variants were reported based on the average of five times of the independent tenfold cross-validation runs.

| **S645** | **RMSE** | **MAE** |
| --- | --- | --- |
| MpbPPI (Backb+Sidec+SASA+AA) | **1.537±0.018** | **0.971±0.010** |
| MpbPPI (Backb+SASA+AA) | 1.601±0.015 | 0.994±0.008 |
| MpbPPI (Backb+Sidec+AA) | 1.635±0.010 | 1.058±0.005 |
| MpbPPI (Backb+AA) | 1.715±0.018 | 1.053±0.012 |
| MpbPPI (w/o AA) | 1.540±0.010 | 1.058±0.005 |
| **M1101** | **RMSE** | **MAE** |
| MpbPPI (Backb+Sidec+SASA+AA) | **1.317±0.015** | **0.839±0.008** |
| MpbPPI (Backb+SASA+AA) | 1.387±0.013 | 0.860±0.007 |
| MpbPPI (Backb+Sidec+AA) | 1.394±0.017 | 0.891±0.005 |
| MpbPPI (Backb+AA) | 1.447±0.020 | 0.885±0.006 |
| MpbPPI (w/o AA) | 1.325±0.012 | 0.835±0.009 |
| **S1131** | **RMSE** | **MAE** |
| MpbPPI (Backb+Sidec+SASA+AA) | 1.298±0.006 | 0.943±0.002 |
| MpbPPI (Backb+SASA+AA) | **1.250±0.004** | **0.878±0.004** |
| MpbPPI (Backb+Sidec+AA) | 1.311±0.003 | 0.948±0.004 |
| MpbPPI (Backb+AA) | 1.325±0.005 | 0.936±0.006 |
| MpbPPI (w/o AA) | 1.372±0.010 | 0.963±0.006 |
| **S4169** | **RMSE** | **MAE** |
| MpbPPI (Backb+Sidec+SASA+AA) | 1.088±0.004 | 0.737±0.001 |
| MpbPPI (Backb+SASA+AA) | **1.073±0.004** | **0.717±0.003** |
| MpbPPI (Backb+Sidec+AA) | 1.120±0.005 | 0.762±0.002 |
| MpbPPI (Backb+AA) | 1.130±0.005 | 0.755±0.003 |
| MpbPPI (w/o AA) | 1.133±0.007 | 0.752±0.004 |

The bold data indicates the best result under current evaluation metric and dataset.

Supplementary Table 4: The RMSE and MAE results of involved methods under the cross-validation that splits data based on the wild-type protein-protein complex types.

| **S645** | **RMSE** | **MAE** |
| --- | --- | --- |
| MpbPPI (Backb+Sidec+SASA+AA) | **1.765±0.037** | **1.180±0.021** |
| MpbPPI (Backb+SASA+AA) | 1.792±0.033 | 1.186±0.019 |
| GeoPPI | $-$ | $-$ |
| FoldX | 2.637 | 1.600 |
| Flex ddG | 3.842 | 2.250 |
| MM/GBSA | 8.102 | 7.174 |
| **M1101** | **RMSE** | **MAE** |
| MpbPPI (Backb+Sidec+SASA+AA) | **1.931±0.042** | **1.367±0.025** |
| MpbPPI (Backb+SASA+AA) | 1.973±0.046 | 1.376±0.036 |
| GeoPPI | $-$ | $-$ |
| FoldX | 3.429 | 2.278 |
| Flex ddG | 4.494 | 2.875 |
| MM/GBSA | 5.925 | 4.496 |
| **S1131** | **RMSE** | **MAE** |
| MpbPPI (Backb+Sidec+SASA+AA) | **2.450±0.080** | **1.772±0.073** |
| MpbPPI (Backb+SASA+AA) | 2.586±0.063 | 1.808±0.059 |
| GeoPPI | $-$ | $-$ |
| FoldX | 2.661 | 1.776 |
| Flex ddG | 3.505 | 2.346 |
| MM/GBSA | 6.518 | 5.076 |
| **S4169** | **RMSE** | **MAE** |
| MpbPPI (Backb+Sidec+SASA+AA) | 1.636±0.009 | 1.068±0.017 |
| MpbPPI (Backb+SASA+AA) | 1.682±0.017 | 1.086±0.019 |
| GeoPPI | 1.899±0.009 | 1.272±0.019 |
| FoldX | 2.263 | 1.461 |
| Flex ddG | 4.488 | 2.972 |
| MM/GBSA | 4.962 | 3.909 |

The bold data indicates the best result under current evaluation metric and dataset.


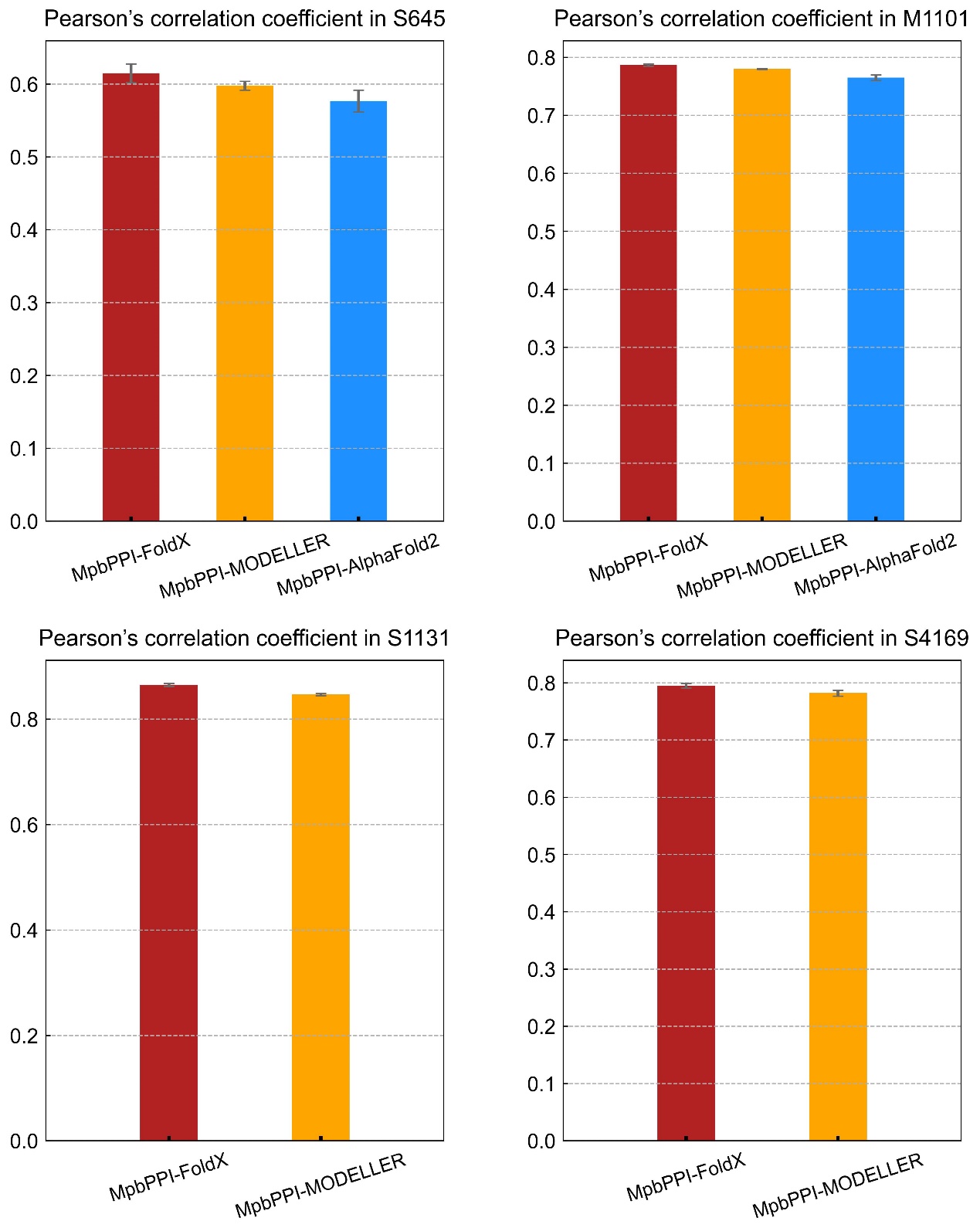


Supplementary Figure 1: The $R_{P}$ results of MpbPPI under the mutation-level tenfold cross-validation on the four PPI $\Delta\Delta G$ datasets.


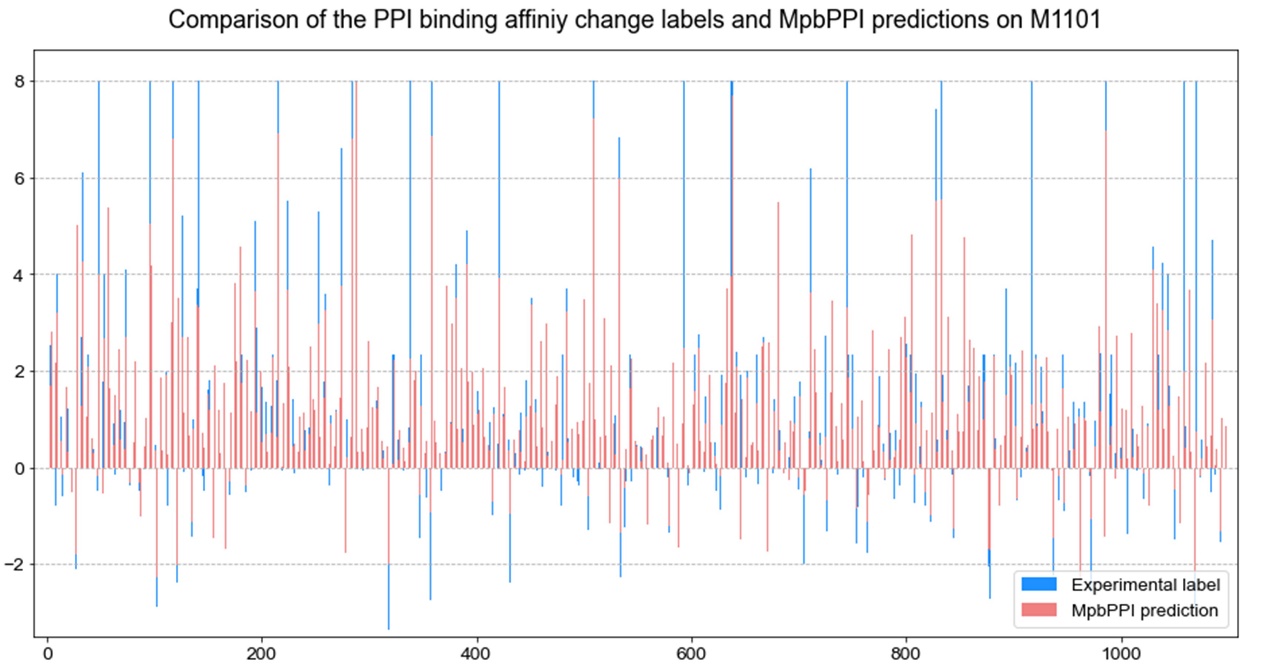


Supplementary Figure 2: the test set prediction results of MpbPPI-FoldX under the mutation-level tenfold cross-validation on M1101 (unit: kcal·mol^-1^). The red bars are the model predictions while the blue ones are corresponding experimental PPI $\Delta\Delta G$ ground truths. Taking the predictions on M1101 as an example, we observed that, MpbPPI-FoldX can successfully give most of the predictions a correct positive or negative sign, i.e., correct binding affinity increase or decrease. In addition, for the outlier samples (with $\Delta\Delta G$ equaling to 8 kcal·mol^-1^), the prediction accuracy is relatively low and consequently influences the overall accuracy. We thought a contributing reason is that such outliers are different from other ground truths and the sample number of them is relatively small, leading to the difficulty of model fitting.
